# Speciation Study on O-Phosphorylethanolamine and O-Phosphorylcholine: Acid–Base Behavior and Mg^2+^ Interaction

**DOI:** 10.3389/fchem.2022.864648

**Published:** 2022-03-28

**Authors:** Donatella Aiello, Massimiliano Cordaro, Anna Napoli, Claudia Foti, Ottavia Giuffrè

**Affiliations:** ^1^ Dipartimento di Chimica e Tecnologie Chimiche, Università Della Calabria, Arcavacata di Rende (CS), Italy; ^2^ Dipartimento di Scienze Chimiche, Biologiche, Farmaceutiche ed Ambientali, Università di Messina, Messina, Italy; ^3^ CNR-ITAE, Messina, Italy

**Keywords:** Mg^2+^, speciation, ligands of biological interest, sequestration, potentiometry, ^1^H-NMR spectroscopy, mass spectrometry, thermodynamic parameters

## Abstract

In the present study, the acid–base behavior of compounds constituting the headgroups of biomembranes, O-phosphorylethanolamine (**PEA**), and O-phosphorylcholine (**PPC**) was investigated by potentiometric titrations in NaCl aqueous solutions at different temperatures (15 ≤ *t*/°C ≤ 37) and ionic strength (0.15 ≤ *I*/mol L^−1^ ≤ 1) values. The complexation properties and the speciation of these ligands with Mg^2+^ were defined under different temperatures (15 ≤ *t*/°C ≤ 37) and *I* = 0.15 mol L^−1^. The results evidenced the formation of three species for **PEA**, namely, MLH_2_, MLH, and ML and two species for **PPC**, namely, MLH and ML. ^1^H-NMR titrations were performed on solutions containing ligand and metal–ligand solutions at *t* = 25°C and *I* = 0.15 mol L^−1^. The estimated values of ligand protonation and complex formation constants and the speciation model are in accordance with the potentiometric data. The enthalpy changes were also determined at *t* = 25°C and *I* = 0.15 mol L^−1^ by the dependence of formation constants on the temperature, confirming the electrostatic nature of the interactions. Matrix-assisted laser desorption mass spectrometry (MALDI-MS) was applied for the characterization of Mg^2+^-L systems (L = **PEA** or **PCC**). MS/MS spectra of free ligands and of Mg^2+^-L species were obtained. The observed fragmentation patterns of both Mg^2+^-L systems allowed elucidating the interaction mechanism that occurs *via* the phosphate group generating a four-membered cycle.

## Introduction

Phospholipids can perform various biological functions ( Takeda et al., 2019 ). For example, phosphatidylcholine plays a fundamental role in the absorption of dietary lipids ( Kennelly et al., 2018 ), phosphatidylglycerol (PG) and phosphatidylinositol (PI) exert antiviral functions against respiratory syncytial virus infection ( Numata et al., 2010; Numata et al., 2015; Takeda et al., 2019 ). More specifically, in mammalian liver cells, one of these two headgroups are contained in two-thirds of the lipids of the plasma membrane, nuclear membrane, mitochondria, microsomes, and Golgi ( Woolf and Roux, 1994 ). The physical state of phospholipid bilayer membranes, as temperature and hydration level are varied, depends to a great extent on the properties of the polar headgroup ( Woolf and Roux, 1994 ). Phospholipids constitute cell membranes and also play other roles as cellular messengers and can perform various biological functions ( Takeda et al., 2019 ). Phosphorylethanolamine (**PEA**) and phosphorylcholine (**PPC**) commonly constitute the headgroups of biological lipid membranes (Gennis, 1989; Woolf and Roux, 1994). **PPC**, a constituent of phosphatidylcholine, is considered as one of the fundamental metabolites in biological systems (Takeda et al., 2019). In mammals, it is synthesized from choline, which is absorbed from food (Fernandez-Botello et al., 2002). Alterations in **PEA** and/or **PPC**, as well as in glycerophosphocholine and glycerophosphoethanolamine, as measured by *in vivo* ^31^ P magnetic resonance spectroscopy in the cerebrospinal fluid (CSF) and subcortical and cortical regions are known to indicate neurodegenerative diseases (Weber-Fahr et al., 2013). In detail, an increase of the **PPC** level in the CSF was observed in patients with Alzheimer’s compared to the normal value of 1.28 μM (Walter et al., 2004). Increased **PEA** levels may indicate inhibition of choline and acetylcholine synthesis (The international standard for identifying health measurements, 2006). Biological membranes are in contact with physiological solutions containing different metal cations. The interactions of the headgroups of lipid membranes with these cations influence their structure and stability (Fukuma et al., 2007; Šegota et al., 2015). Metal complexation is also important in cation transport, lipoprotein formation, and several biochemical processes (Hendrickson and Fullington, 1965).

Among metal cations, magnesium is a main bioelement, together with calcium, sodium, and potassium. Magnesium and calcium are necessary to bind biological macromolecules by using negatively charged components (Nies, 2004). In 1926 Leroy was the first to describe the essential role played by Mg ^2+^ in living organisms. The first investigation of its deficiency in humans was published in 1934 by Hirschfender and Haury ( Vormann, 2004 ). In the following years the lack of Mg ^2+^ has been linked with a series of diseases in humans ( Flink, 1956 ). Since then, the role of magnesium in physiological processes has attracted increasing attention ( Vormann, 2004 ). In biological systems, magnesium is present as Mg^2+^, and being smaller than Ca^2+^, it attracts water molecules more strongly (Saris et al., 2000). The large hydration shell of hydrated magnesium makes it difficult to enter biological membranes by passing through narrow channels (Vormann, 2004). It is the second most abundant cation within the cell. The intracellular free magnesium concentration is approximately 0.5 mmol L^−1^. It is mainly bound to proteins, negatively charged phospholipids, ATP, and nucleic acids (Heaton, 1993). The concentration of magnesium in the plasma is in equilibrium with that adsorbed on the bone surface (Elin, 1994). The magnesium concentration in a healthy adult is as follows: in the erythrocytes, 2.5 mmol L^−1^; in the blood, 0.7–1.1 mmol L^−1^, of which 55% free, 32% bound primarily to albumin, and 13% bound to citrate, phosphate, etc; in the cerebrospinal fluid, 1.25 mmol L^−1^ of which 55% free and 45% complexed; and in the sweat, 0.3 mmol L^−1^ (Shils, 1997; Weisinger and Bellorin-Font, 1998).

The excess magnesium present in the blood is excreted by the kidney. Precisely, the glomerular membrane of the kidney filters about 80% of the total serum magnesium (Quamme and de Rouffignac, 2000). Its high concentration inhibits its reabsorption, causing an increase in its loss from the human body (Dai et al., 2001). In adult humans, the dietary magnesium intake was set at 300–420 mg per day (The National Academies, 1997; Deutsche Gesellschaft für Ernährung, 2000). The main extracellular effects of the magnesium cation are represented by its ability to crosslink the negatively charged phospholipids in the membranes, stabilizing the latter and at the same time reducing their fluidity (Flatmann, 1993). One of the main features of Mg^2+^ is the high charge density, greater than other ions in the cells, so that its involvement with high negative charge density compounds, such as phosphate and pyrophosphate metabolites, prevails (Frausto da Silva and Williams, 2001).

**FIGURE 1:**
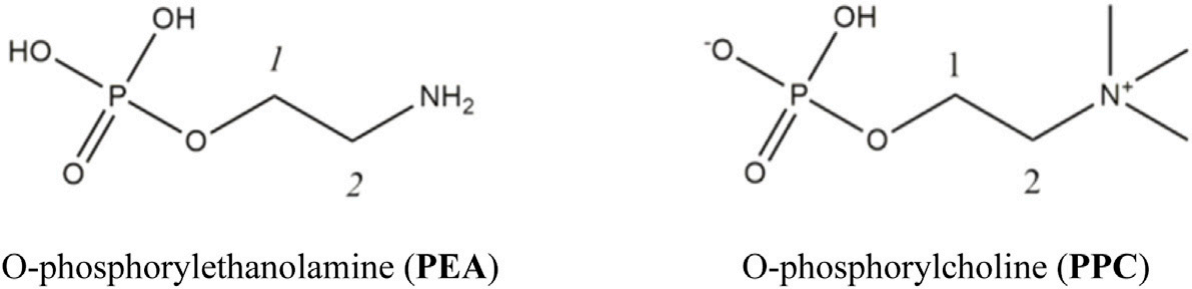
Ligands under study.

In this study, the interaction between Mg^2+^ ions and two phosphoryl compounds present in biological membranes, i.e., **PEA** and **PPC**, represented in Figure 1, has been elucidated *via* a multidisciplinary approach. The aim was to evaluate the strength of the interaction by potentiometry and ^1^H-NMR spectroscopy and to explain the mechanism by MALDI mass spectrometry and MS/MS. The determination of reliable thermodynamic data is necessary to simulate distribution of species in biological fluid conditions and, therefore, to assess biological phenomena such as transport through membranes but also for evaluating the possible use of these compounds in some application fields. Indeed, **PPC** is employed in biomaterials for clinical applications (Matsuura et al., 2016; Goda and Miyahara, 2018), and it is well known that the performance of these biomaterials can be affected by electrolytes (Wu et al., 2018; Díaz-Betancor et al., 2019). Therefore, the speciation studies can be crucial evaluating the performance of these compound-based biomaterials after interaction with Mg^2+^.

## Materials and Methods

### Materials

O-phosphorylethanolamine and O-phosphorylcholine chloride solutions were prepared by weighing and subsequent dissolution of the corresponding products (Sigma-Aldrich/Merck, Darmstadt, Germany). The purity of the ligands, determined by alkalimetric titration, was greater than 99%. Magnesium chloride solutions were prepared by weighing and dissolving the Fluka (Fluka/Honeywell, Charlotte, North Carolina, United States) product. These solutions were standardized using the EDTA (Ehylenediaminetetraacetic acid disodium salt, BioUltra, ≥99%) standard.

Sodium chloride solutions were obtained by weighing the salt (puriss., Sigma-Aldrich/Merck, Darmstadt, Germany), after drying at 110°C. The solutions of hydrochloric acid and sodium hydroxide were obtained by diluting the Fluka (Fluka/Honeywell, Charlotte, North Carolina, United States) vials. Subsequently, they were standardized by titrations using sodium carbonate (≥99.5%, Sigma-Aldrich/Merck, Darmstadt, Germany) and potassium acid phthalate (≥99.5%, Sigma-Aldrich Merck, Darmstadt, Germany), respectively. These salts were previously dried in an oven at 110°C.

### Potentiometric Apparatus and Procedure

Two distinct potentiometric systems were used for titrations. Each system has an identical configuration with a Metrohm model 809 Titrando potentiometer, an automatic dispenser Metrohm Dosino 800, and a Metrohm LL-Unitrode WOC combined glass electrode. A PC was connected to each potentiometric system to acquire experimental titration data by Metrohm TIAMO 2.2 software. Several parameters, such as the titrant delivery and e.m.f. stability, were controlled by this software. Estimated accuracies of these systems are ±0.15 mV and ±0.002 ml for e.m.f. and for titrant volumes, respectively.

For the ligand protonation, for each titration, volumes of the NaOH standard were added to 25 ml of the solution containing **PEA** or **PPC** at C_L_ = 5–10 mmol L^−1^, 0.15 ≤ *I*/mol L^−1^ ≤ 1 in NaCl at *t* = 25°C, and *I* = 0.15 mol L^−1^ at *t* = 15, 37°C. For metal–ligand complexes, 25 ml of the solution containing Mg^2+^ and **PEA** or **PPC** at C_M_ = 1–4 mmol L^−1^, C_L_ = 2–4 mmol L^−1^, C_M_/C_L_ = 0.33-2, and *I* = 0.15 mol L^−1^ in NaCl was titrated by using the NaOH standard at *t* = 15, 25, and 37°C. All the solutions during the titrations were in glass jacket thermostated cells, under magnetic stirring and by bubbling pure N_2_. Independent titrations of HCl with standard NaOH were performed to obtain the values of the standard electrode potential E^0^ and pK_w_, under the same ionic strength and temperature conditions of the corresponding measurement.

### NMR Apparatus and Procedure

A Varian NMR spectrometer 500 Mhz was used to process ^1^H-NMR spectra. 1,4-dioxane was used as the internal reference (*δ*_CHdioxane_ = 3.70 ppm), and all chemical shifts refer to tetramethylsilane (TMS). All measurements were carried out using the presaturation technique to reduce the water signal, in 9:1 H_2_O/D_2_O solution at *t* = 25°C. The spectra containing ligands **PEA** or **PPC** (at C_L_ = 7 mmol L^−1^) and NaCl (*I* = 0.15 mol L^−1^) solutions were recorded in a pH range between 2 and 11. The spectra containing **PEA** or **PPC** and Mg^2+^ (C_M_ = 6 mmol L^−1^, C_L_ = 7 mmol L^−1^) and NaCl solutions were recorded in the same pH range of the free ligands.

### Mass Spectrometric Apparatus and Procedure

All metal complexes were prepared, as published elsewhere (Chillè et al., 2020; Aiello et al., 2021a). Briefly, all ligands (1 or 2 mmol) were dissolved in 100 μl of water; the pH was adjusted to 8 with NaOH and then added to an aqueous solution (200 μL) of MgCl_2_ (1 mmol). The resulting solutions were maintained under magnetic stirring, at room temperature for 2 h. MALDI mass spectrometry analysis was performed on a 1 μl portion of a premixed solution containing the reaction mixture and the matrix α-CHCA (0.3% in TFA), in a 2:10 (v:v) ratio.

All MS and MS/MS experiments were performed, as published elsewhere (Aiello et al., 2018; Imbrogno et al., 2019). All experiments were conducted using a 5800 MALDI-TOF/TOF analyzer (AB-SCIEX), supplied with a neodymium–yttrium–aluminum–garnet laser, operating at 349 nm. MS spectra were obtained with a mass accuracy of 5 ppm, by collecting 4,000 laser shots, applying a laser pulse rate of 400 Hz. A total of 5,000 laser shots, at a pulse rate of 1000 Hz and 1 kV of collision energy, were collected and averaged for each MS/MS experiment. Δppm of the MS/MS experiments was 20 ppm. MS/MS experiments were achieved using ambient air as the collision gas (10^–6^ Torr). Data Explorer (version 4.0) was used for handling all spectra.

### Calculations

The STACO and BSTAC programs were employed to process the experimental potentiometric data. With their use, the protonation constants of the ligands, the formation constants of the complexes, and the parameters of the acid–base titration (the standard potential E^0^, junction potential, and analytical concentration of the reagents) were obtained. The LIANA program was used in processing experimental results at various ionic strengths and temperatures to obtain the dependence of protonation and formation constants on ionic strength and temperature. More information about BSTAC, STACO, and LIANA can be found in the reference (De Stefano et al., 1997). The speciation diagrams and the percentages of complex species were obtained using the HySS program (Alderighi et al., 1999). HypNMR software was used to process the observed experimental signals, assuming a fast mutual exchange in the NMR time scale (Frassineti et al., 1995). With its use, the protonation constants of **PEA** and **PPC**, the formation constants of the complex species, and the individual chemical shifts of each species were calculated.

## Results and Discussion

### Acid–Base Behavior, Complexation With Mg^2+^, and Speciation Profiles

The protonation constants of the two ligands under study, **PEA** and **PPC**, necessary for the subsequent determination of the complexes with Mg^2+^, were determined. The protonation reactions as overall formation constants (β) and stepwise formation constants (K) are as follows, where the charges are omitted for simplicity:

$${\text{L}+\text{rH }=\text{ LH}}_{\text{r}}\;\;\;\;\;\;\;\;\;\;\;\;\;\;\;\;\;\;\;{\text{β}}^{{\text{LH}}_{\text{r}}},$$ (1)

$${\text{LH}}_{\text{r}-1}{\;+\text{ H }=\text{ LH}}_{\text{r}}\;\;\;\;\;\;\;\;\;\;\;\;\;\;\;\;\;\;\;K^{{\text{LH}}_{\text{r}}}.$$ (2)

Protonation constant values obtained *via* potentiometric titrations under different temperature and ionic strength conditions are summarized in Table 1. The calculated values referred to **PEA** at *I* = 0.15 mol L^−1^ and *t* = 25°C (log*K* ^LH^ = 10.141, log*K* ^LH2^ = 5.590) are similar to those reported by Mohan *et al.*, log*K* ^LH^ = 10.12, log*K* ^LH2^ = 5.52 (at *I* = 0.2 mol L^−1^, *t* = 25°C in KNO_3_) (Mohan and Abbott, 1978a; Mohan and Abbott, 1978b). In a very recent study, the protonation constants of **PEA** at *t* = 20°C and *I* = 0.1 mol L^−1^ in KNO_3_ were proposed (log*K* ^LH^ = 10.41, log*K* ^LH2^ = 5.70) (Gabryel-Skrodzka et al., 2021). It is not possible to make other comparisons at other temperatures or ionic strengths since in the literature, there are only data up to 0.2 mol L^−1^ and *t* = 20 or 25°C (Datta and Grzybowski, 1959; Wozniak and Nowogrocki, 1979; May and Murray, 2001; Pettit and Powell, 2001; Martell et al., 2004). As far as we know, in the literature, there are no thermodynamic parameters on the protonation of **PPC**.

**TABLE 1:** Experimental values of protonation constants of PEA and PPC and formation constants of Mg^2+^ species obtained by potentiometry at different temperatures and ionic strength values in NaCl.

L	Species			logβ^H^ ^a^		
		*t* = 15°C	*t* = 25°C	*t* = 25°C	*t* = 25°C	*t* = 37°C
		*I* = 0.15^b^	*I* = 0.15^b^	*I* = 0.5^b^	*I* = 0.90^b^	*I* = 0.15^b^
PEA	LH	10.381(2)^c^	10.141(2)^c^	10.071(4)^c^	10.087(2)^c^	9.836(7)^c^
	LH_2_	16.021(3)	15.731(4)	15.607(7)	15.551(4)	15.560(9)
	LH_3_	17.08(3)	16.69(3)	16.79(2)	16.45(4)	17.29(2)
	MLH_2_	17.78(4)	17.29(3)	—	—	16.96(9)
	MLH	12.61(2)	11.56(6)	—	—	11.65(7)
	ML	2.79(2)	2.66(3)	—	—	1.94(6)
PPC	LH	5.635(3)^c^	5.646(4)^c^	5.542(2)^c^	5.459(4)^c^	5.668(4)^c^
	LH_2_	—	6.53(3)	6.23(2)	6.12(3)	6.71(3)
	MLH	6.79(3)	7.46(3)	—	—	8.07(3)
	ML	1.62(2)	1.42(6)	—	—	2.24(3)
				**log*K* ^H^ ^d^**		
PEA	LH	10.381	10.141	10.071	10.087	9.836
	LH_2_	5.640	5.590	5.536	5.464	5.724
	LH_3_	1.06	0.96	1.18	0.90	1.73
	MLH_2_	1.76	1.56	—	—	1.40
	MLH	2.23	1.41	—	—	1.81
	ML	2.79	2.66	—	—	1.94
PPC	LH	5.635	5.646	5.542	5.459	5.668
	LH_2_	—	0.88	0.69	0.66	1.04
	MLH	5.17	6.04	—	—	5.83
	ML	1.62	1.42	—	—	2.24

a Overall protonation or formation constants.

b In mol L^−1^.

c ≥95% of confidence interval.

d Stepwise protonation or formation constants.

The species with Mg^2+^ were subsequently investigated. Both the protonation constants reported in this study and the hydrolysis constant of Mg^2+^, reported in the Supplementary Table S1 under various conditions, were considered. Potentiometric experimental titrations were carried out at different metal/ligand ratios and different concentrations, to select the most reliable speciation model and to obtain the formation constants of the complex species. These Mg^2+^(M)-ligand(L) formation constants are indicated as overall formation constants (β) or stepwise formation constants (*K*), based on the following reactions, where the charges are omitted for simplicity:

$$\text{M}+\text{L}+\text{rH}={\text{MLH}}_{r}\;\;\;\;\;\;\;\;\;\;\;\;\;\;\;\;\;\;\;\;{\text{β}}^{{\text{MLH}}_{\text{r}}},$$ (3)

$$\text{M}+{\text{LH}}_{r}={\text{MLH}}_{r}\;\;\;\;\;\;\;\;\;\;\;\;\;\;\;\;\;\;\;\;K^{{\text{MLH}}_{\text{r}}}.$$ (4)

The choice of the speciation model that best reflects the system under study is made considering some requirements such as its simplicity, goodness of statistical parameters (standard and mean deviations referring to the fit), percentages of formation of complex species, variance ratio between the chosen model, and others (Filella and May, 2005).

**FIGURE 2:**
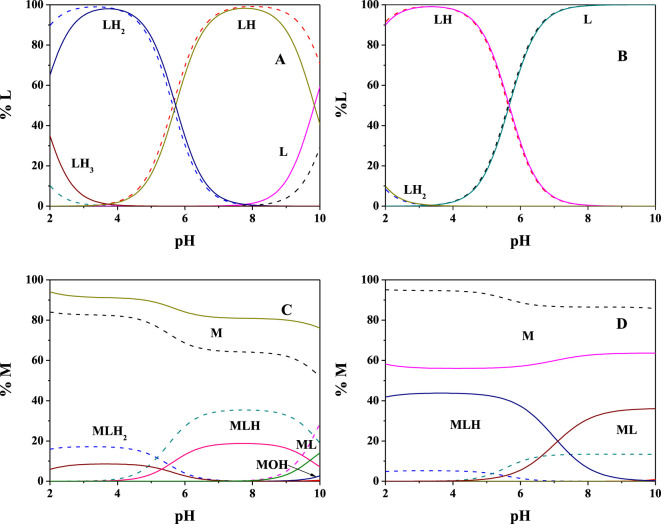
Speciation diagrams at *t* = 15°C (dotted lines) and *t* = 37°C (solid lines) for **(A)** L = **PEA, (B)** L = **PPC** (C_L_ = 5 mmol L^−1^ and *I* = 0.15 mol L^−1^ in NaCl); **(C)** Mg^2+^(M)-**PEA**(L), and **(D)** Mg^2+^(M)-**PPC**(L) (C_M_ = 2 mmol L^−1^, C_L_ = 4 mmol L^−1^, and *I* = 0.15 mol L^−1^ in NaCl).

The obtained results, in terms of formation constants of Mg^2+^-**PEA** and Mg^2+^-**PPC** species at *I* = 0.15 mol L^−1^ in NaCl and *t* = 15, 25, and 37°C, are reported in Table 1. The speciation models include three species for the Mg^2+^-**PEA** system, namely, MLH_2_, MLH, and ML and two species for the Mg^2+^-**PPC** system, namely, MLH and ML. Mass spectrometry measurements will also highlight the formation of ML_2_ species. Despite the excess ligand employed in the experimental potentiometric conditions (M:L = 1:3), the formation percentage of ML_2_ species was negligible for both ligands. Therefore, this species was not considered in the speciation models. The speciation diagrams of the systems containing **PEA**, **PPC**, Mg^2+^-**PEA**, and Mg^2+^-**PPC** are shown in Figures 2A–D. Under physiological conditions (pH = 7.4, *t* = 37°C, and *I* = 0.15 mol L^−1^), considering **PEA** at C_L_ = 5 mmol L^−1^, formation percentages of L, LH, and LH_2_ species are 0.3, 97.4, and 2.2, respectively. Under the same conditions, considering **PPC**, formation percentages of L and LH species are 98.3 and 1.7, respectively (Figure 2B). In the presence of Mg^2+^, in the **PEA** system, MLH species achieves a significant formation percentage of 18.6 (Figure 2C); in the **PPC** system, both MLH and ML species achieve significant formation percentages equal to 15.3 and 23.7, respectively (Figure 2D). More in detail, Figure 2C—referring to the Mg^2+^-**PEA** system—shows that in the acid pH range, the MLH_2_ species is formed, reaching percentages of up to 10%. The main species is MLH with 20% in the pH range 6.5–9.0. ML species is formed at pH > 9. With regard to the Mg^2+^-**PPC** system, shown in Figure 2D, the observed complex species are much higher than those of **PEA** under the same conditions. MLH species exceeds 40% at pH = 2–4. ML species reaches almost 40% at pH = 7–10. In the literature, fairly close to our results of formation constants of Mg-**PEA** species at *I* = 0.15 mol L^−1^ and *t* = 25°C were reported by Hendrickson *et al.*, log*K* ^ML^ = 2.20 and log*K* ^MLH^ = 1.48 (at *I* = 0.1 mol L^−1^ in (C_3_H_7_)_4_NI) and *t* = 20°C) (Hendrickson and Fullington, 1965). Other values were obtained by Mohan *et al.*, log*K* ^ML^ = 1.56 and log*K* ^MLH^ = 1.17 (at *I* = 0.2 mol L^−1^ in KNO_3_ and *t* = 25°C) (Mohan and Abbott, 1978a; Mohan and Abbott, 1978b) and by Osterberg, log*K* ^ML^ = 1.70 and log*K* ^MLH^ = 1.23 (at *I* = 0.15 mol L^−1^ in KCl and *t* = 25°C) (Osterberg, 1960). It was not possible to compare the results reported in this study on Mg^2+^-**PPC** species with those in the literature since as far as we know, the speciation patterns and the formation constants were not reported up to now.

**FIGURE 3:**
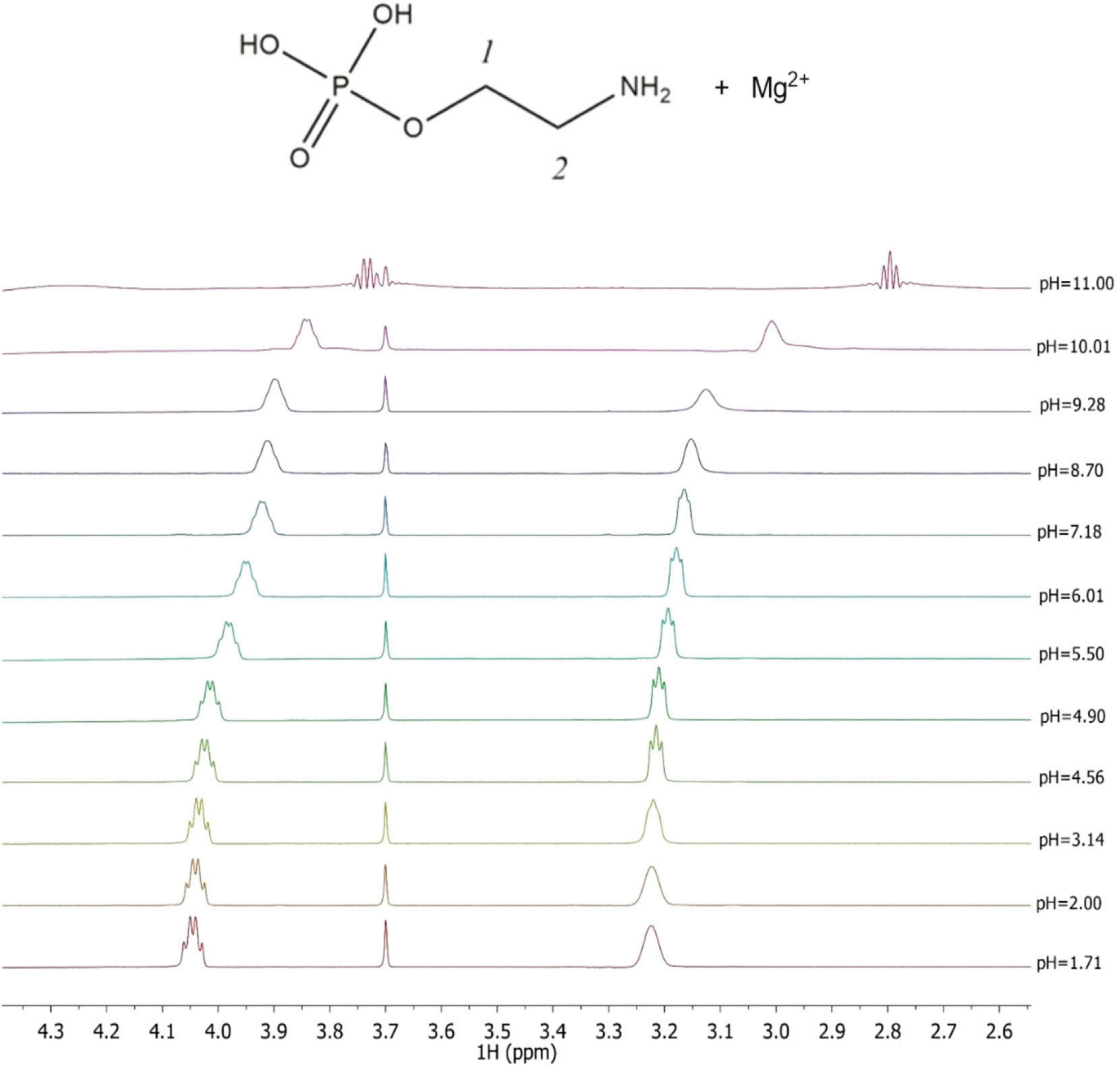
Superimposed ^1^H-NMR spectra of solutions containing Mg^2+^(M) e **PEA**(L) at C_M_ = 6 mmol L^−1^, C_L_ = 7 mmol L^−1^, *t* = 25°C, and *I* = 0.15 mol L^−1^ in NaCl, range of pH = 1.71–11.00.

^1^H-NMR titrations were also carried out at *I* = 0.15 mol L^−1^ and *t* = 25°C both for the determination of the protonation constants of the ligands under study as well as of the complexes with Mg^2+^, as already reported on other systems (Cardiano et al., 2011a; Cardiano et al., 2017). The Chemical shift and pattern of protons of the **PEA** solution are shown in Supplementary Figure S1 at different pH values. Some noticeable signals can be identified for the two types of **PEA** protons: a doublet triplet (td) assignable to the CH_2_ protons in position 1 showing Δδ = 0.28 ppm from pH 1.67 to pH 11.00 and a triplet (t) assignable to the CH_2_ protons in position 2 showing Δδ = 0.43 ppm from pH 1.67 to pH 11.00. The solutions of **PEA** with Mg^2+^ were analyzed in the same pH range by the NMR technique, and the proton spectra are shown in Figure 3. The data collected indicate that the chemical shift values have the same trend as the values recorded for the free ligand, as evidenced in the graph showing small differences (Supplementary Figure S2). Therefore, it can be assumed that the interaction between the metal and ligand occurs from the phosphoric moiety or by the electrostatic interaction of the negative oxygen atoms and the magnesium cation.

**FIGURE 4:**
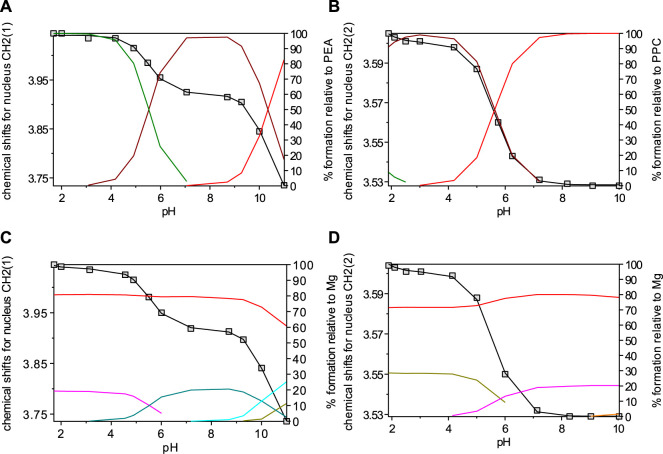
Overlap of calculated (line) and observed (□) chemical shifts with speciation diagrams obtained by HypNMR on solutions at *t* = 25°C and *I* = 0.15 mol L^−1^ containing: **(A)** PEA at C_PEA_ = 7.6 mmol L^−1^ (Lines: green, LH_2_; brown, LH; red, L); **(B)** PPC at C_PPC_ = 7 mmol L^−1^ (Lines: green, LH_2_; brown, LH; red, L); **(C)** Mg^2+^-PEA at C_M_ = 6 mmol L^−1^ and C_PEA_ = 7 mmol L^−1^ (Lines: red, free M; violet, MLH_2_; green, MLH; light blue, ML; dark green, MOH); **(D)** Mg^2+^-PPC at C_M_ = 6 mmol L^−1^ and C_PPC_ = 7 mmol L^−1^ (Lines: red, free M; dark green, MLH; violet ML).

The chemical shift and pattern of protons of the **PPC** solution are shown in Supplementary Figure S3 at different pH values. Some noticeable chemical shifts can be identified for the three types of **PPC** protons: a broad multiplet (m) assignable to the CH_2_ protons in position 1 showing Δδ = 0.14 ppm from pH 1.90 to pH 10.00, a triplet (t) assignable to the CH_2_ protons in position 2 showing Δδ = 0.13 ppm from pH 1.90 to pH 10.00, and a singlet of the nine methyl protons at 3.16 ppm, which does not have appreciable variations, in the pH range studied. The solutions of **PPC** with Mg^2+^ were analyzed by the NMR technique, and the proton spectra are shown in Supplementary Figure S4. Also, for **PPC** solutions, the chemical shift values of the free ligand and the ligand with magnesium are similar, evidencing small differences, as shown in the graph (Supplementary Figure S5). A similar assumption on **PEA** and **PPC** could also interact through the phosphate group oxygen, and this would justify the lack of chemical shift of the protons on the aliphatic chain of the ligand in the presence of magnesium ions. The results listed in Table 2, together with those obtained by potentiometry, were obtained from the processing of the measured chemical shifts. These results confirmed that the comparison between the results obtained with the two techniques, under the same experimental conditions, shows an excellent agreement both for the speciation model determined by potentiometry and for the values of the formation constants of the complexes, especially for **PPC**. Figure 4 highlights the excellent agreement between the experimental and the calculated chemical shift values over all pH ranges considered and therefore in the formation areas of the different species.

**TABLE 2:** Comparison between the experimental protonation constants of **PEA** and **PPC** and experimental formation constants of Mg^2+^-**PEA** and Mg^2+^-**PPC** species obtained *via* ^1^H-NMR and potentiometry at *t* = 25°C and *I* = 0.15 mol L^−1^.

Ligand	Species	logβ^a^	
		^1^H-NMR	Potentiometry
PEA	LH	10.32(2)^b^	10.141
	LH_2_	15.88(5)	15.731
	LH_3_	16.69^c^	16.69
	MLH_2_	17.29^c^	17.29
	MLH	11.78(8)	11.56
	ML	2.85(6)	2.66
PPC	LH	5.64(1)^b^	5.646
	LH_2_	6.53^c^	6.53
	MLH	7.36(4)	7.46
	ML	1.44(7)	1.42

a Overall protonation constants.

b ≥95% of confidence interval.

c These values, obtained by potentiometry, were kept constant during the calculations with HypNMR.

### Simulation in Biological Fluids

One of the aims of this investigation is to be able to use the acquired thermodynamic information making simulations under real fluid conditions, such as biological ones. Just as an example, the composition of two biological fluids was considered with the aim of evaluating the significance of the complex species under study for the purposes of a characterization of the fluid itself. Among the biological fluids, the cerebrospinal fluid (CSF) and the extracellular fluid in the brain intracellular space were considered. The CSF is formed in the brain. It is an aqueous solution containing higher contents of magnesium, sodium, and chloride and lower concentrations of potassium, calcium, bicarbonate, and phosphate, with respect to the plasma in humans (Artru, 2010). The latter in the brain plays a role in multiple key functions, including non-synaptic neurotransmission. The extracellular space makes up about 15% of the total brain volume and is filled with an extracellular fluid whose electrolyte composition differs enough from that of the cerebrospinal fluid (Heinemann et al., 2009).

**FIGURE 5:**
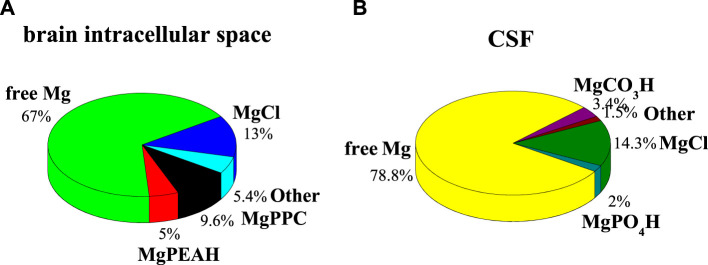
Calculated percentages of Mg^2+^-**PEA** and Mg^2+^-**PPC** species in biological fluids at pH = 7.4, *t* = 37°C, and *I* = 0.15 mol L^−1^. **(A)** Brain intracellular space conditions; **(B)** CSF conditions.

The complexes that they form with Mg ^2+^ can be relevant and not negligible for the purposes of a characterization of the fluid itself. One example regards the calculation of the formation percentages of Mg^2+^-**PEA** and -**PPC** species under CSF conditions (C_Na_ = 141 mmol L^−1^, C_K_ = 2.9 mmol L^−1^; C_Ca_ = 1.25 μmol L^−1^, C_Mg_ = 1.2 μmol L^−1^, C_Cl_ = 124 mmol L^−1^, C_HCO3_ = 21 mmol L^−1^, C_PO4_ = 0.15 mmol L^−1^, C_PEA_ = 1.70 μmol L^−1^, C_PPC_ = 1.70 μmol L^−1^, *t* = 37°C, and *I* = 0.15 mol L^−1^) (Artru, 2010). All formation constants of the species taken into account in these simulations are listed in Supplementary Table S3. Under these conditions, none of the species containing **PEA** and **PPC** reaches significant formation percentages. Calculated percentages of Mg^2+^-**PEA** and -**PPC** species in CSF conditions at pH = 7.4, *t* = 37°C, and *I* = 0.15 mol L^−1^ are shown in Figure 5B. On the contrary, considering the conditions of the brain intracellular space, where **PEA** and **PPC** concentrations are higher than in the CSF and plasma (C_Na_ = 155 mmol L^−1^, C_K_ = 3.0 mmol L^−1^; C_Ca_ = 1.6 mmol L^−1^, C_Mg_ = 1.2 mmol L^−1^, C_Cl_ = 135 mmol L^−1^, C_HCO3_ = 21 mmol L^−1^, C_PO4_ = 1.0 mmol L^−1^, C_PEA_ = 0.59 mmol L^−1^, C_PPC_ = 0.59 mmol L^−1^, *t* = 37°C, *I* = 0.15 mol L^−1^ (Klein et al., 1993; Eaton and Pooler, 2009; Barrett et al., 2013), the formation percentages, especially of the Mg^2+^-**PPC** species, increase significantly. More in detail, free magnesium reaches 67%, MgCl 13%, Mg**PPC** 9.6%, and Mg**PEA**H 5%. Calculated percentages of Mg^2+^-**PEA** and -**PPC** species, under the conditions of the extracellular fluid in the brain intracellular space, at pH = 7.4, *t* = 37°C, and *I* = 0.15 mol L^−1^, are shown in Figure 5A. This result shows that the species Mg**PPC** reaches a percentage not negligible, albeit not high. The availability of reliable thermodynamic constants makes simulation possible under the conditions of real fluids.

### Dependence of Formation Constants on Temperature and Ionic Strength

The dependence of protonation constants and formation constants on ionic strength was determined processing experimental measurements performed at different ionic strengths by considering the following Debye–Huckel equation, widely used in the 0 ≤ *I* ≤ 1 mol L^−1^ range (Cardiano et al., 2018b; Chillè et al., 2018):

$$\text{logβ}=\log\beta^{0}\mathrm{- 0}\mathrm{.51}\cdot \mathrm{z*}\frac{\sqrt{I}}{1+1\mathrm{.5}\sqrt{I}}+\;C\;\mathrm{I,}$$ (5)

where β is the stability constant at a given ionic strength, β^0^ is the stability constant at infinite dilution, z* = Σ(charge)^2^_reactants_ − Σ(charge)^2^_products_, and *C* is an empirical parameter. Protonation constants at infinite dilution and C parameter values calculated by Eq. 5 for **PEA** and **PPC** species at *t* = 25°C in NaCl are listed in Table 3.

**TABLE 3:** Protonation constants at infinite dilution and parameters for the dependence on ionic strength Eq. 5, of PEA and PPC species at *t* = 25°C in NaCl.

Ligand	Species	logβ^0^ ^a^	*C*
PEA	LH	10.59(3)^b^	0.33(5)^b^
	LH_2_	16.43(5)	0.36(9)
	LH_3_	17.5(1)	0.2(2)
PPC	LH	6.14(3)^b^	0.14(9)^b^
	LH_2_	7.27(3)	0.06(4)

a Overall protonation constants.

b ≥95% of confidence interval.

For the dependence on the temperature, the van’t Hoff equation was used, as for other systems (Cordaro et al., 2019; Giuffrè et al., 2020):

$$\text{log}\beta^{\text{T}}\;=\text{ log}\beta^{\theta }+\text{Δ}H\left(\;1/\theta -1/T\right)\text{Rln}10\text{,}$$ (6)

where logβ^T^ is the stability constant at a given ionic strength and temperature (in Kelvin), logβ^θ^ is the value at the reference temperature (*T* = 298 K), and Δ*H* ^0^ is the formation enthalpy change expressed in kJ mol^−1^ at *T* = 298.15 K and R = 8.314472 J K^−1^ mol^−1^.

**FIGURE 6:**
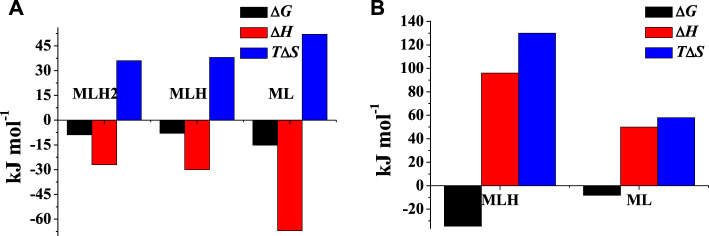
Formation thermodynamic parameters of **(A)** Mg^2+^-**PEA** and **(B)** Mg^2+^-**PPC** species at *t* = 25°C and *I* = 0.15 mol L^−1^ in NaCl.

The values of thermodynamic parameters, as formation enthalpy, entropy, and free energy changes, of the Mg^2+^-**PEA**, and -**PPC** species are reported in Table 4. Formation thermodynamic parameters referring to reaction (4) are shown as a bar plot in Figure 6, to highlight the prevalent contribution of entropy or enthalpy to free energy. As known for electrostatic interactions, the entropic term, related to the orientation disorder of the solvation water molecules, constitutes the main contribution to the free energy change. This behavior was found for most of the species, except for ML one formed by the **PEA** ligand.

**TABLE 4:** Thermodynamic parameters of protonation of PEA and PPC and of formation of Mg^2+^-PEA and Mg^2+^-PPC species at *t* = 25°C and *I* = 0.15 mol L^−1^ in NaCl.

Ligand	Species	Δ*G* ^a,b^	Δ*H* ^a,b^	*T*Δ*S* ^a,b^
PEA	LH	−57.9	−42(1)^c^	16
	LH_2_	−31.9	6(2)	38
	LH_3_	−5.5	54(5)	59
	MLH_2_	−8.9	−27(2)	36
	MLH	−8.0	−30(10)	38
	ML	−15.2	−67(10)	52
PPC	LH	−32.2	3(4)^c^	35
	LH_2_	−5.0	45(5)	50
	MLH	−34.5	96(3)	130
	ML	−8.1	50(8)	58

a Referred to stepwise protonation and formation constants.

b Expressed in kJ mol^−1^.

c ≥95% of confidence interval.

### Sequestering Ability

The sequestering capacity of a ligand in a solution correlates with the tendency of the ligand to form complexes with a given metal cation. The higher the stability of the complex species, the lower is the concentration of the free metal cation in the solution. To evaluate the sequestering ability of a ligand toward a specific metal cation, all equilibria in which both the ligand and the metal cation participate are considered, as metal ion hydrolysis, ligand protonation, and weak interactions with the ionic medium. The pL_0.5_ empirical parameter, i.e., the co-logarithm of the ligand concentration which sequesters 50% of the metal cation in traces, was proposed. Traces of metal cations were considered as they represent the conditions of concentration with which many of them are generally present in natural fluids. The sequestering capacity of a ligand toward a metal cation can be evaluated by the following Boltzmann-type equation with asymptotes 0 for pL → 0, 1 for pL→∞ (Cardiano et al., 2011b; Cardiano et al., 2018a):

$$\chi \;=\frac{1}{1+10^{\left(\text{pL}–{\text{pL}}_{0.5}\right)}},$$ (7)

where χ is the sum of the molar fractions of the metal–ligand species, and pL is the co-logarithm of the total ligand concentration. The sequestering ability strictly depends on pH, temperature, and ionic strength.

**FIGURE 7:**
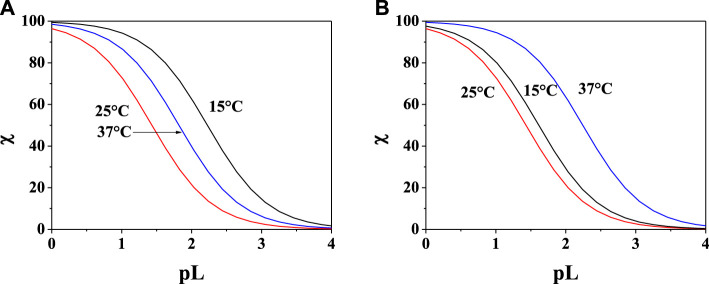
Sum of the fractions of **(A)** Mg^2+^-**PEA** and **(B)** Mg^2+^-**PPC** species at pH = 7.4, and *I* = 0.15 mol L^−1^ in NaCl.

In order to evaluate the sequestering capacity of **PEA** and **PPC** toward Mg^2+^, pL_0.5_ values at different temperatures were calculated (Supplementary Table S4). Figures 7A,B show the comparison between the sequestering capacity of **PEA** and **PPC** under physiological conditions (pH = 7.4, and *I* = 0.15 mol L^−1^). The plot confirms that under these conditions, **PPC** shows a slightly higher sequestering capacity than **PEA** toward Mg^2+^.

### Mass Spectrometry

Mass spectrometry measurements were used to elucidate the mechanism of **PPC** and **PEA** interactions. In turn, this aspect is very useful to understand some biological phenomena such as the mechanism transport through membranes but also for evaluating the possible use of these compounds in some application fields. Phosphorylcholine-based biomaterials are well studied due to their biocompatibility and being used in many clinical applications (Matsuura et al., 2016; Goda and Miyahara, 2018). The performance of these biomaterials can be affected by electrolytes. In fact, the zwitterionic nature of the phosphorylcholine groups may be changed into a cationic system introducing divalent cations that strongly interact with the phosphate group. Although it required the knowledge of the sequestering ability, the coordination mode of the natural ligand in local microenvironments helps to understand the potential activity of the biomaterials, and speciation studies are seldom reported (Gabryel-Skrodzka et al., 2021).

**FIGURE 8:**
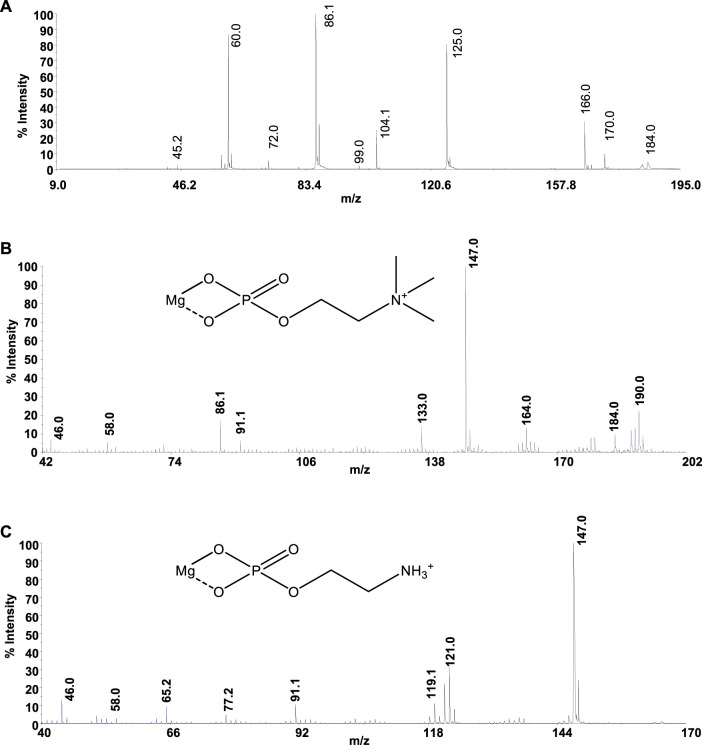
Product-ion spectra of **(A)** [**PPC**]^+^, **(B)** [Mg**PPC**]^+^, and **(C)** [Mg**PEA**]^+^.

Mass spectrometry (MS) techniques are generally used for the highly sensitive analysis of metal ion complexes. The positive ion mode is usually the polarity for metal complex analysis by mass spectrometry (Aiello et al., 2017). Here, this selection was strengthened using ligands containing a quaternary nitrogen with a fixed positive charge. Therefore, the matrix-assisted laser desorption mass spectrometry (MALDI-TOF/TOF-MS) platform in the positive ion mode was adopted to study Mg^2+^-L systems (L = **PPC** or **PEA**). This platform offers particular advantages in investigating biological systems (Aiello et al., 2021b). The most important peculiarities of MALDI MS-based methods rely in the rapid and sensitive detection of analytes (Aiello et al., 2020; Salvatore et al., 2020) and in obtaining the molecular profiling of complex mixtures. Finally, the structures of low molecular weight organic and organometallic compounds can be analyzed and determined by tandem mass spectrometry (MS/MS) experiments (Falcone et al., 2013). The interaction of Mg^2+^ with **PPC** and **PEA** was explored by MALDI using α–CHCA as the matrix. Full-scan positive ion MS of Mg^2+^-L systems (L = **PPC**, or **PEA**) displayed ion species indicating information on ML_n_ (n: 1, 2) species. The elemental composition of detected ML_n_ species, combined with the observed gas-phase fragmentation pathways, was used to identify the coordination sites and to ascribe the most probable structures of complexes. The isotope ratio patterns observed for all the Mg^2+^/L complexes matched with those obtained from theoretical calculations, suggesting both ligands acting as a bidentate. The simplest systems, represented by free ligands, will first be discussed (Figure 8A). The MS/MS spectrum of [**PPC**H]^+^ at 1 kV shows complementary fragment ion pairs of the m/z values 99/86 ([H_4_O_4_P]^+^/[C_5_H_12_N]^+^), 81/104 ([H_2_O_3_P]^+^/[C_5_H_14_NO]+), and 60/125 ([C_3_H_10_N]^+^/[C_2_H_6_O_4_P]^+^) as the most abundant fragment ions. **PPC** is an aliphatic ester of phosphoric acid; consequently, it preferentially forms the fragment of m/z 99 rather than the phosphate marker ion of m/z 81. Meanwhile, an intramolecular H transfer, involving the alkyl backbone of the **PPC** molecule, promotes the release of trimethylamine and the formation of the m/z 125 ([C_2_H_6_O_4_P]^+^). MS and MS/MS ion fragments of ligands are listed in Table 5.

**TABLE 5:** MS and MS/MS ion fragments of ligands and Mg^2+^ species.

PPC	Composition	Detected	PEA	Composition	Detected
	[C_5_H_15_NO_4_P]^+^	184.07	—	[C_2_H_9_NO_4_P]^+^	142.03
	[C_4_H_13_NO_4_P]^+^	170.06	—	[CH_6_O_4_P]^+^	113.00
	[C_5_H_13_NO_3_P]^+^	166.06	—	[C_2_H_6_O_4_P]^+^	125.00
	[C_2_H_6_O_4_P]^+^	125.00	—	[H_4_O_4_P]^+^	98.99
	[C_5_H_14_NO]^+^	104.11	—	[H_4_O_3_P]^+^	82.99
	[H_4_O_4_P]^+^	98.99	—	—	—
	[C_5_H_12_N]^+^	86.10	—	—	—
	[H_4_O_3_P]^+^	82.99	—	—	—
	[C_4_H_10_N]^+^	72.08	—	—	—
	[C_3_H_10_N]^+^	60.08	—	—	—
MS [Mg**PPC**]^+^	[C_5_H_13_MgNO_4_P]^+^	206.04	MS [Mg(**PPC**)_2_]^+^	[C_10_H_27_MgN_2_O_8_P_2_]^+^	389.11
MS/MS	[C_5_H_15_NO_4_P]^+^	184.08	MS/MS	[C_7_H_18_MgNO_8_P_2_]^+^	330.04
	[C_2_H_4_MgO_4_P]^+^	146.97	—	[C_4_H_9_MgO_8_P_2_]^+^	270.97
	[C_2_H_7_MgNO_4_P]^+^	164.00	—	[C_2_H_8_MgNO_7_P_2_]^+^	243.96
	[C_5_H_14_NO]^+^	104.11	—	[H_5_MgO_8_P_2_]^+^	218.93
	[CH_2_MgO_4_P]^+^	132.96	—	[C_7_H_19_MgN_2_O_8_P_2_]^+^	345.05
	[C_5_H_12_N]+	86.10	—	[C_4_H_12_MgNO_8_P_2_]^+^	287.99
	[C_4_H_10_N]^+^	72.08	—	[C_5_H_13_MgNO_4_P]^+^	206.05
	[C_3_H_8_N]^+^	58.08	—	[H_3_MgO_7_P_2_]+	200.92
	[C_2_H_8_N]^+^	46.07	—	[C_2_H_6_O_4_P]^+^	125.00
	[C_4_H_9_MgNO_4_P]^+^	190.02	—	[C_5_H_14_NO]+	104.11
	[CH_4_MgO_4_P]^+^	134.97	—	[C_5_H_12_N]+	86.10
	—	—	—	[C_4_H_10_N]^+^	72.08
	—	—	—	[C_3_H8N]^+^	58.08
MS [Mg**PEA**]^+^	[C_2_H_7_MgNO_4_P]^+^	164.00	MS [Mg(**PEA**)_2_]^+^	[C_4_H_15_MgN_2_O_8_P2]^+^	305.02
	[CH_4_MgO_4_P]^+^	134.97	—	[C_4_H_12_MgNO_8_P2]^+^	287.99
	[H_2_MgO_4_P]^+^	120.96	—	[C_2_H_10_MgNO_8_P_2_]^+^	261.98
	[C_2_H_4_MgO_4_P]^+^	146.97	—	[C_2_H_5_MgO_6_P_2_]^+^	210.94
	[MgO_3_P]^+^	102.94	—	[C_2_H_4_MgO_4_P]^+^	146.97
	[H_2_O_3_P]^+^	80.98	—	[C_2_H_4_O4P]^+^	122.99
	[C_2_H_4_O_2_P]^+^	91.00	—	[C_2_H_4_O2P]^+^	91.00
	[C_2_H_4_NO]^+^	58.03	—	[C_2_H_4_NO]^+^	58.03
	[C_2_H_8_N]^+^	46.07	—	—	—

Direct MS analysis of solutions containing both Mg^2+^ and **PPC** at a final M:L ratio of 1:1, pH 8 as stated by speciation experiments, showed the formation of the ions of m/z 206 ([Mg**PPC**]^+^, [C_5_H_13_MgNO_4_P]^+^) and m/z 389 ([Mg(**PPC**)_2_]^+^, and [C_10_H_27_MgN_2_O_8_P_2_]^+^). As shown in Figure 8B and Table 5, the product-ion spectrum of the [Mg**PPC**]^+^ (m/z 206) contains prominent ions of m/z 147 ([C_2_H_4_MgO_4_P]^+^) and m/z 91 ([C_2_H_4_O_2_P]^+^). The pathways proposed for formation of these two ions involve an initial loss of trimethylamine ([MgL-59]), followed by an additional loss of Mg(OH)_2_ (58 Da). Meanwhile, the observed daughter ion of m/z 191 ([C_4_H_10_MgNO_4_P]^•+^), 177 ([C_3_H_8_MgNO_4_P]^•+^), and 163 ([C_2_H_6_MgNO_4_P]^•+^) arises from direct and consecutive loss of methyl groups matching typical fragmentation of the positively-charged head group. The gas-phase behavior of [Mg(**PEA**)H]^+^ matches that observed for Mg/**PPC** systems (Figure 8C and Table 5). The observed fragmentation patterns of both Mg systems enable us to design the most probable molecular structures, where the coordination occurs at the phosphate group generating a four-membered cycle.

**FIGURE 9:**
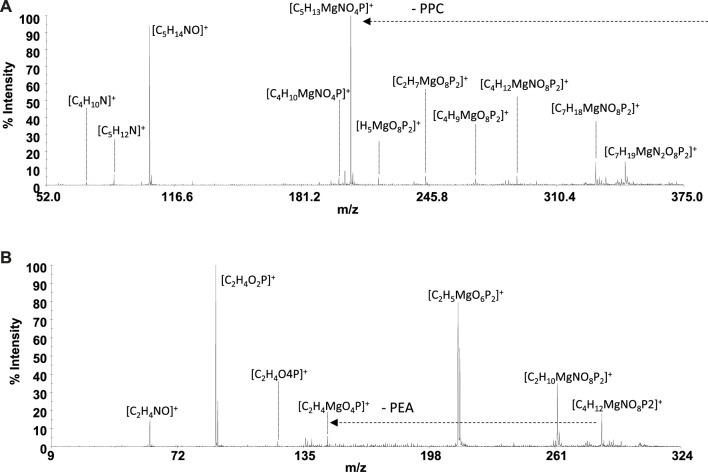
Product-ion spectra of **(A)** [Mg(**PPC)**_2_]^+^ and **(B)** [Mg(**PEA)**_2_]^+^.

Despite the experimental potentiometric conditions, the significant formation of MgL_2_ species was not found; to complete this study, a solution containing Mg^2+^:L (L = **PPC** or **PEA**), at a final ratio of 1:2, was analyzed by MALDI MS/MS spectrometry at different times, over 2 h. The experiments conducted allowed a better detection (% intensity of the total ion current) of [MgL_2_]^+^ ions. The MALDI MS/MS spectrum of the [Mg(**PPC**)_2_]^+^ (m/z 389 and [C_10_H_27_MgN_2_O_8_P_2_]^+^ Figure 9A and Table 5) revealed the formation of the cations [C_5_H_13_MgNO_4_P]^+^, [H_3_MgO_7_P_2_]^+^, [C_2_H_6_O_4_P]^+^, [C_5_H_14_NO]^+^, [C_5_H_12_N]^+^, [C_4_H_10_N]^+^, and [C_3_H_8_N]^+^, resulting from the initial loss of the ligand, followed by the additional fragmentations of [Mg**PPC**]^+^. Furthermore, the direct loss of trimethylamine [Mg(**PPC**)_2_-59] (m/z 330), followed by the additional loss of trimethylamine [Mg(**PPC**)-118]^+^(m/z 270) and/or ethylene (28) [Mg(**PPC**)_2_-(145)]^+^ from the parent ion [Mg(**PPC**)_2_]^+^, confirmed that the phosphate group to be involved in the coordination with Mg^2+^. Interestingly, the loss of one ligand molecule from the [M(**PPC**)_2_]^+^ species was observed. These data indicated that coordination of Mg^2+^ with **PPC** causes weakening of specific bonds which break upon collision. The gas-phase behavior of [Mg(**PEA**)_2_]^+^ does not match that observed for Mg/**PPC** systems. For this species, the direct loss aminoethanol ([Mg(**PEA**)_2_-60], m/244) followed by the additional loss of the methylamine ([Mg(**PEA**)_2_-93]^+^, m/212) dominates the gas-phase fragmentation (Figure 9B and Table 5). The formation of [Mg(**PEA**)-17]^+^ suggests that coordination of Mg(II) with **PEA** is more rugged than **PPC** under MS/MS conditions. The ML and ML_2_ complexes of Mg^2+^ with **PEA** features coordination modes which were very similar to those observed for the species containing **PPC**. For all species detected, mass spectra suggested a common structure in which metal is coordinated to the phosphate group of the ligand frame.

## Conclusion

The interactions between molecules constituting the headgroups of biological lipid membranes, such as **PEA** and **PCC** with metal cations present in biological fluids, are key factors as they can modify physicochemical properties, structure, and cell functioning. Any modification in membrane composition may significantly affect its physicochemical properties, structure, and cell function. As an example, modification in membrane composition in nerve cells is characteristic of neurodegenerative diseases. Therefore, the elucidation of the acid–base behavior of **PEA** and **PPC** and their complexing capacities toward cations of physiological relevance can assume crucial importance. As an example, the zwitterionic nature of the phosphorylcholine group in **PPC**-based biomaterials, employed in many clinical applications, can also be changed with the introduction of a metal cation. In particular, the coordination mode of **PPC** with metal cations contributes to understand the potential activity of the biomaterials. For all these reasons, a multidisciplinary study was undertaken to elucidate the interaction between **PEA** and **PPC** with Mg^2+^, one of the main bioelements. The study described here offers useful information necessary for the interpretation of the nature of the metal–ligand interaction. Thanks to the assessment of reliable thermodynamic data, it was possible to calculate the sequestering ability of the ligands under study toward Mg^2+^ and also to make simulations under the conditions of biological fluids. For example, the results of simulations carried out under conditions of the extracellular fluid in the brain intracellular space showed that Mg**PCC** achieves a non-negligible percentage of formation. MALDI-MS and MS/MS were employed for the characterization of the free **PPC** and **PEA** ligands and of their interactions with Mg^2+^, both never investigated until now. The observed fragmentation pathways of both Mg^2+^-L systems suggested a common interaction mechanism in which the metal is coordinated to the phosphate group of the ligand frame, giving rise to a four-membered cycle.

## Data Availability

The original contributions presented in the study are included in the article/Supplementary Material, further inquiries can be directed to the corresponding author.
