# Supplementary material for: Speciation Study on O-Phosphorylethanolamine and O-Phosphorylcholine: Acid–Base Behavior and Mg2+ Interaction
Source: Front Chem. 2022 Mar 28;10:864648. doi: 10.3389/fchem.2022.864648 (PMC8996081; doi:10.3389/fchem.2022.864648)
Supplement: Supplementary file 1 [file DataSheet1.docx]

**Supplementary Information**

**Supplementary Table S1.** Hydrolysis Constants of Mg^2+^ at different temperatures and ionic strength (in mol L^-1^)

| Reaction |  |  | logβ ^a)^ |  |  | Ref. |
| --- | --- | --- | --- | --- | --- | --- |
|  | *t* = 15°C  *I* = 0.15 | *I* = 0.15 | *t* = 25°C  *I* = 0.5 | *I* = 1 | *t* = 37°C  *I* = 0.15 |  |
| Mg^2+^ + H_2_O = Mg(OH)^+^ + H^+^ | -11.99 | -11.73 | -11.74 | -11.68 | -11.45 | ^a)^ |

^a)^ Crea, F.; De Stefano, C., Milea, D., Pettignano, A., Sammartano, S. (2015). SALMO and S3M: A Saliva Model and a Single Saliva Salt Model for Equilibrium Studies, Bioinorg. Chem. Appl. 2015.

**Supplementary Table S2**. Calculated chemical shifts (in ppm) of protons of **PEA** and **PPC** species at *t* = 25°C, *I* = 0.15 mol L^-1^ in NaCl

| Ligand |  | L | LH | LH_2_ | LH_3_ | MLH_2_ | MLH | ML |
| --- | --- | --- | --- | --- | --- | --- | --- | --- |
| **PEA** | **δ_CH2(1)_** | 3.695(2)^a)^ | 3.922(2)^a)^ | 4.038(2)^a)^ | 4.109(2)^a)^ | 4.037(1)^a)^ | 3.915(1)^a)^ | 3.719(1)^a)^ |
|  | **δ_CH2(2)_** | 2.711(6) | 3.171(6) | 3.220(6) | 3.217(6) | 3.222(1) | 3.157(1) | 2.767(1) |
| **PPC** | **δ_CH2(1)_** | 4.100(1) | 4.235(1) | 4.438(1) | ⎯ | ⎯ | 4.232(1) | 4.114(1) |
|  | **δ_CH2(2)_** | 3.529(1) | 3.600(1) | 3.734(1) | ⎯ | ⎯ | 3.601(3) | 3.533(3) |

^a )^ ≥95% confidence interval.

**Supplementary Table S3**. Formation constant values of the species considered for simulations under CSF and extracellular fluid in the brain intracellular space (*t* = 25°C, *I* = 0.15 mol L^-1^)

| Reaction | logβ | Ref. |
| --- | --- | --- |
| H^+^ + OH^-^ = H_2_O | -13.45 | ^a)^ |
| Mg^2+^ + H_2_O = Mg(OH)^+^ + H^+^ | -11.45 | ^a)^ |
| Ca^2+^ + H_2_O = Ca(OH)^+^ + H^+^ | -12.56 | ^a)^ |
| **PEA**^2-^ + H^+^ = PEAH^-^ | 9.836 | This paper |
| **PEA**^2-^ + 2H^+^ = PEAH_2_^0^ | 15.560 | This paper |
| **PEA**^2-^ + 3H^+^ = PEAH_3_^+^ | 17.29 | This paper |
| **PPC**^2-^ + H^+^ = PPCH^-^ | 5.668 | This paper |
| **PPC**^2-^ + 2H^+^ = PPCH_2_^0^ | 6.71 | This paper |
| Mg^2+^ + **PEA**^2-^+2H^+^ = MgPEAH_2_^2+^ | 16.96 | This paper |
| Mg^2+^ + **PEA**^2-^+H^+^ = MgPEAH^+^ | 11.65 | This paper |
| Mg^2+^ + **PEA**^2-^ = MgPEA^0^ | 1.94 | This paper |
| Mg^2+^ + **PPC**^2-^+H^+^ = MgPPCH^+^ | 8.07 | This paper |
| Mg^2+^ + **PPC**^2-^ = MgPPC^0^ | 2.24 | This paper |
| PO_4_^3-^ + H^+^ = HPO_4_^2-^ | 11.64 | ^a)^ |
| PO_4_^3-^ + 2H^+^ = H_2_PO_4_^-^ | 18.47 | ^a)^ |
| PO_4_^3-^ + 3H^+^ = H_3_PO_4_^0^ | 20.50 | ^a)^ |
| Mg^2+^ + PO_4_^3-^ + H^+^ = MgHPO_4_^0^ | 13.72 | ^a)^ |
| Mg^2+^ + PO_4_^3-^ + 2H^+^ = MgH_2_PO_4_^+^ | 19.67 | ^a)^ |
| Ca^2+^ + PO_4_^3-^ + H^+^ = CaHPO_4_^0^ | 13.58 | ^a)^ |
| Ca^2+^ + PO_4_^3-^ + 2H^+^ = CaH_2_PO_4_^+^ | 19.54 | ^a)^ |
| Na^+^ + PO_4_^3-^ = NaPO_4_^2-^ | 0.95 | ^a)^ |
| Na^+^ + PO_4_^3-^ + H^+^ = NaHPO_4_^-^ | 12.41 | ^a)^ |
| Na^+^ + PO_4_^3-^ + 2H^+^ = NaH_2_PO_4_^0^ | 18.69 | ^a)^ |
| 2Na^+^ + PO_4_^3-^ = Na_2_PO_4_^-^ | 1.75 | ^a)^ |
| 2Na^+^ + PO_4_^3-^ + H^+^ = Na_2_HPO_4_^0^ | 12.13 | ^a)^ |
| K^+^ + PO_4_^3-^ = KPO_4_^2-^ | 0.85 | ^a)^ |
| K^+^ + PO_4_^3-^ + H^+^ = KHPO_4_^-^ | 12.22 | ^a)^ |
| K^+^ + PO_4_^3-^ + 2H^+^ = KH_2_PO_4_^0^ | 18.49 | ^a)^ |
| 2K^+^ + PO_4_^3-^ = K_2_PO_4_^-^ | 1.39 | ^a)^ |
| 2K^+^ + PO_4_^3-^ + H^+^ = K_2_HPO_4_^0^ | 12.16 | ^a)^ |
| Na^+^ + K^+^ + PO_4_^3-^ = NaKPO_4_^-^ | 1.93 | ^a)^ |
| Na^+^ + K^+^ + PO_4_^3-^ + H^+^ = NaKHPO_4_^0^ | 12.45 | ^a)^ |
| CO_3_^2-^ + H^+^ = HCO_3_^-^ | 9.85 | ^a)^ |
| CO_3_^2-^ + 2H^+^ = H_2_CO_3_^0^ | 15.97 | ^a)^ |
| Mg^2+^ + CO_3_^2-^ = MgCO_3_^0^ | 2.22 | ^a)^ |
| Mg^2+^ + CO_3_^2-^ + H^+^ = MgHCO_3_^+^ | 10.56 | ^a)^ |
| Ca^2+^ + CO_3_^2-^ = CaCO_3_^0^ | 2.56 | ^a)^ |
| Ca^2+^ + CO_3_^2-^ + H^+^ = CaHCO_3_^+^ | 10.86 | ^a)^ |
| Na^+^ + CO_3_^2-^ = NaCO_3_^-^ | 0.80 | ^a)^ |
| Na^+^ + CO_3_^2-^ + H^+^ = NaHCO_3_^0^ | 9.87 | ^a)^ |
| K^+^ + CO_3_^2-^ = KCO_3_^-^ | 0.61 | ^a)^ |
| K^+^ + CO_3_^2-^ + H^+^ = KHCO_3_^0^ | 9.79 | ^a)^ |
| Mg^2+^ + Cl^-^ = MgCl^+^ | 0.18 | ^a)^ |
| Ca^2+^ + Cl^-^ = CaCl^+^ | 0.03 | ^a)^ |
| Na^+^ + Cl^-^ = CaCl^0^ | -0.5 | ^a)^ |
| K^+^ + Cl^-^ = KCl^0^ | -0.48 | ^a)^ |

^a)^ Crea, F.; De Stefano, C., Milea, D., Pettignano, A., Sammartano, S. (2015). SALMO and S3M: A Saliva Model and a Single Saliva Salt Model for Equilibrium Studies, Bioinorg. Chem. Appl. 2015.

**Supplementary Table S4**. pL_0.5_ values of **PEA** e **PPC** towards Mg^2+^ at different temperatures values, pH = 7.4, *I* = 0.15 mol L^-1^ in NaCl

| Ligand | *t* /°C | pL_0.5_ |
| --- | --- | --- |
| **PEA** | 15 | 2.23 |
|  | 25 | 1.43 |
|  | 37 | 1.82 |
| **PPC** | 15 | 1.62 |
|  | 25 | 1.43 |
|  | 37 | 2.24 |


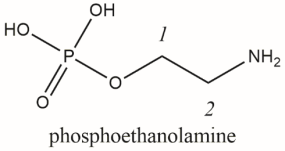


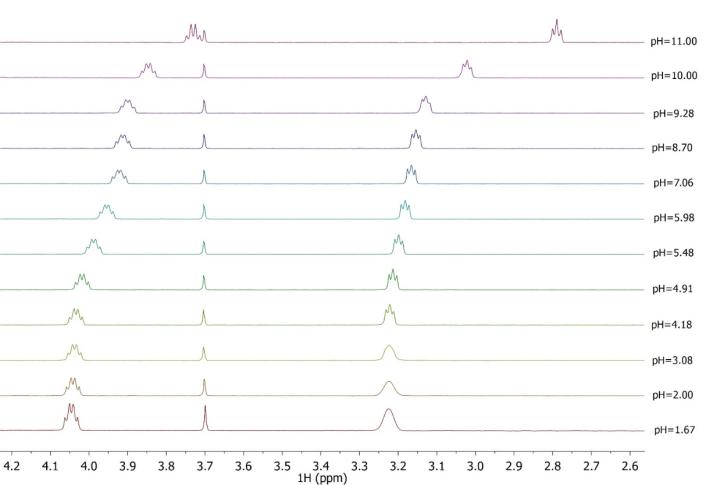


**Supplementary Figure S1**. Superimposed ^1^H-NMR spectra of solutions containing **PEA** at C = 7.6 mmol L^-1^, *t* = 25°C, *I* = 0.15 mol L^-1^ in NaCl, range of pH = 1.67-11.00.

**Supplementary Figure S2.** Comparison of chemical shift values of methylene protons 1 e 2 on solutions containing **PEA** and **PEA** with Mg^2+^.


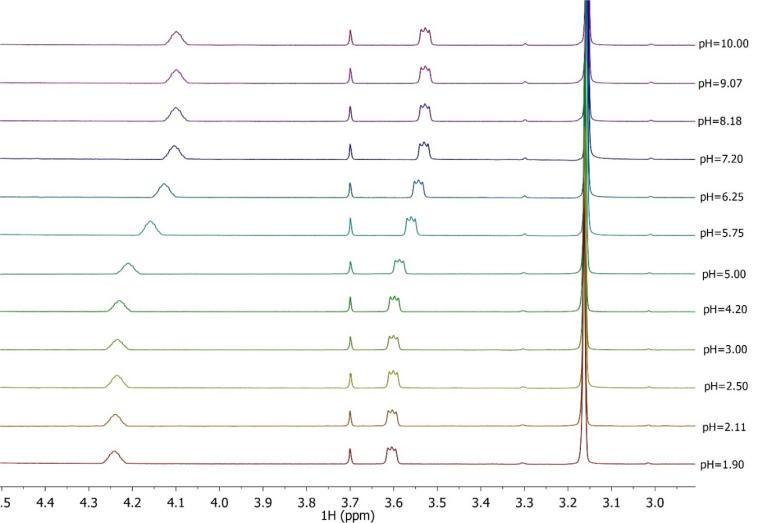

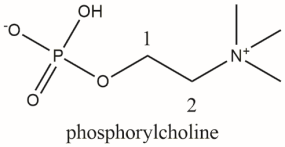


**Supplementary Figure S3.** Superimposed ^1^H-NMR spectra of solutions containing **PPC** at C = 7 mmol L^-1^, *t* = 25°C, *I* = 0.15 mol L^-1^ in NaCl, range of pH = 1.90-10.00.


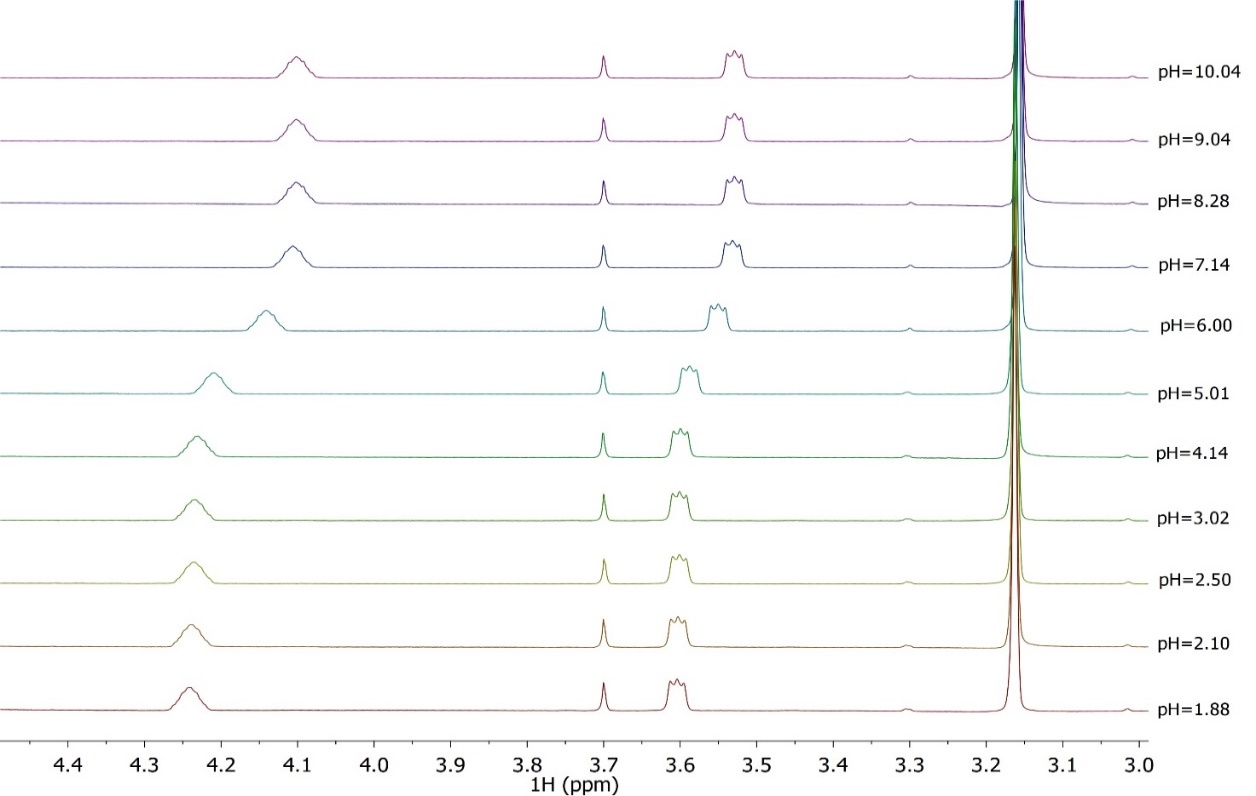

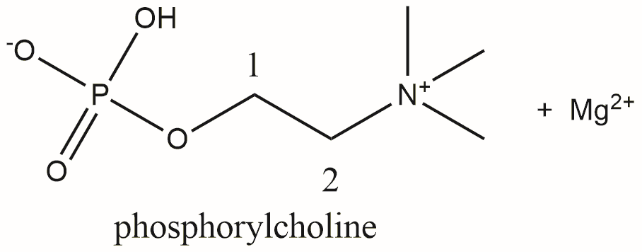


**Supplementary Figure S4.** Superimposed ^1^H-NMR spectra of solutions containing Mg^2+^(M) and **PPC**(L) at C_M_ = 6 mmol L^-1^, C_L_ = 7 mmol L^-1^, *t* = 25°C, *I* = 0.15 mol L^-1^ in NaCl, range of pH = 1.88-10.04.


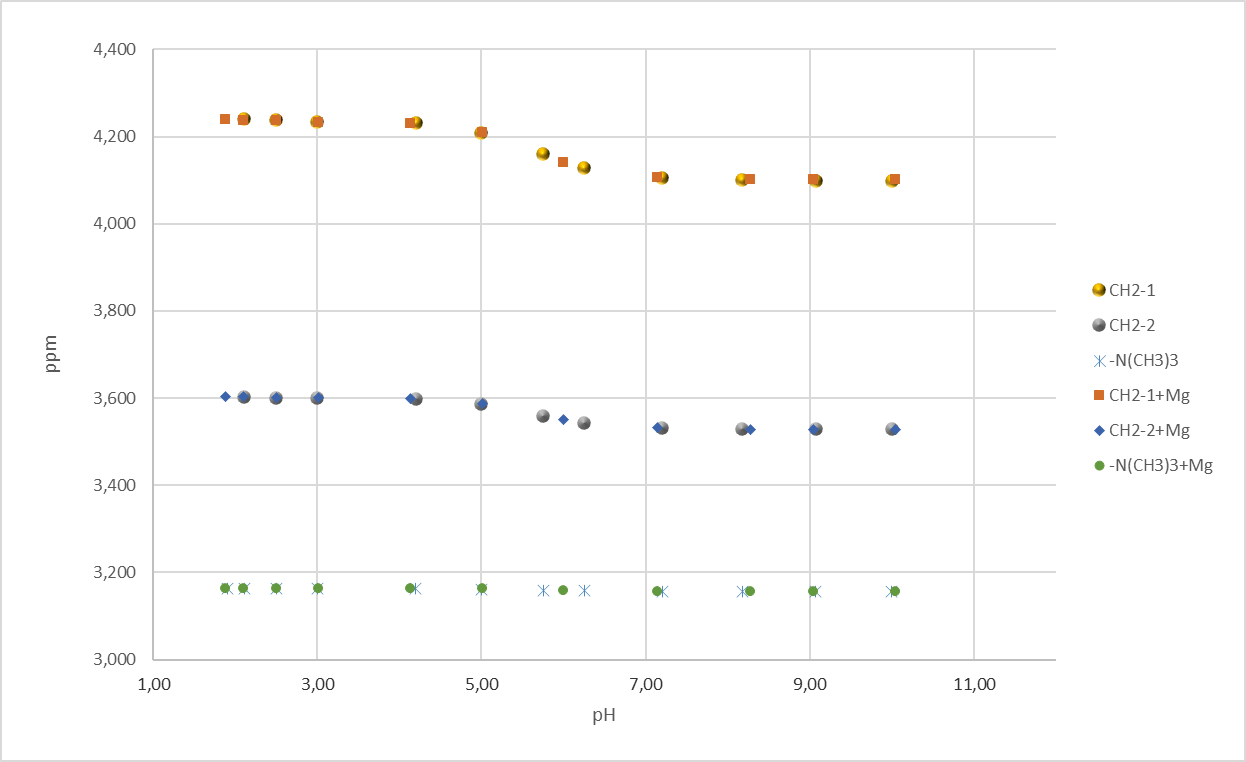


**Supplementary Figure S5**. Comparison of chemical shift values of methylene protons 1 e 2 on solutions containing **PPC** and **PPC** with Mg^2+^.
